# Magnetic resonance imaging for translational and basic life sciences

**DOI:** 10.1186/1479-5876-10-S2-A29

**Published:** 2012-10-17

**Authors:** Ed X Wu

**Affiliations:** 1 Department of Electrical and Electronic Engineering, the University of Hong Kong, Hong Kong SAR, China; 2 Laboratory of Biomedical Imaging and Signal Processing, the University of Hong Kong, Hong Kong SAR, China; 3 Medical Engineering Program, the University of Hong Kong, Hong Kong SAR, China

## Background

With advances in engineering and computing, an extraordinary body of imaging technologies and applications has developed over the last 35 years. One of the most important applications of such technologies is the study of anatomy, physiology, pathology and functions in humans and animal models of human development and diseases. Among the various *in vivo* and *non-invasive* imaging modalities available or under development today, magnetic resonance imaging (MRI) is the most powerful and versatile technology platform. Its unparalleled in vivo and quantitative capabilities offer a broad range of applications covering from noninvasive morphologic measurements, tissue microstructural characterization, hemodynamic and vascular characterization, metabolite measurements, sub-system physiologies, brain functions to monitoring of cell migrational dynamics. This presentation will illustrate these technological developments with some of the ongoing rodent brain MRI projects in our laboratory, highlighting the capacity of MRI as a platform technology to visualize the central nervous system (CNS) in vivo from molecules to systems levels. They include diffusion characterization of neural tissue microstructure; functional study of molecular pathways by spectroscopy; functional study of brain development and injury; monitoring of endogenous neural stem cell activities; and novel contrast agents for brain imaging.

